# Combination Therapy for Reconstructive Periodontal Treatment in the Lower Anterior Area: Clinical Evaluation of a Case Series

**DOI:** 10.3390/dj6040050

**Published:** 2018-10-01

**Authors:** Carlos E. Nemcovsky, Ilan Beitlitum

**Affiliations:** Department of Periodontology and Dental Implantology Dental School, Tel-Aviv University, Tel Aviv 6139001, Israel; beilan@post.tau.ac.il

**Keywords:** periodontal regeneration, connective tissue graft, enamel matrix proteins derivative, root coverage, combination therapy, soft tissue management, periodontal surgery

## Abstract

Clinically, periodontal regeneration may be achieved by the application of barrier membranes, grafts, wound-healing modifiers, and their combinations. Combination therapy refers to the simultaneous application of various periodontal reconstructive treatment alternatives to obtain additive effects. This approach may lead to assemblage of different regenerative principles, such as conductivity and inductivity, space provision and wound stability, matrix development and cell differentiation. The application of autogenous connective tissue grafts during periodontal regenerative treatment with enamel matrix proteins derivative (EMD) has been previously reported. The present case series present a modified approach for treatment of severe periodontally involved lower incisors presenting with thin gingival biotype, gingival recession, minimal attached and keratinized gingiva width and muscle and/or frenum pull. In all cases a combination therapy consisting of a single buccal access flap, root conditioning, EMD application on the denuded root surfaces and a free connective tissue graft was performed. Clinical and radiographic outcomes were consistently satisfactory, leading to probing depth reduction, clinical attachment gain, minimal gingival recession, increased attached and keratinizing gingival width, elimination of frenum and/or muscle pull together with radiographic bone fill of the defects. It may be concluded that the present combination therapy for reconstructive periodontal treatment in the lower anterior area is a valuable alternative for indicated cases.

## 1. Introduction

Reconstructive periodontal procedures improve tooth survival while reducing periodontitis progression and re-intervention needs providing long-term outcome stability [1]. Provided adequate periodontal treatment and maintenance, even with reduced periodontal attachment level, natural dentition yields better long-term survival and marginal bone level changes compared with dental implants [2].

Periodontal reconstruction is a complex biological process that involves de novo formation of the lost tooth supporting structures, including alveolar bone, periodontal ligament, and cementum over a previously diseased root surface.

Reconstructive periodontal procedures have shown advantages over conventional surgical procedures in terms of better results in long-term stability, improved tooth survival, less periodontitis progression and fewer needs for re-intervention over long periods [1].

In addition, certain delivery agents have been proven to improve the efficacy of non-surgical periodontal therapy [3,4,5]. Clinically, periodontal regeneration may be achieved by application of barrier membranes, grafts, wound-healing modifiers, and their combinations.

Guided tissue regeneration (GTR) is based on the application of a separating barrier membrane mechanically isolating the defect. Although this mechanical/biological concept has been widely proven both in pre-clinical and clinical studies, several shortcomings such as treatment of multiple proximal defects, complications due to membrane exposure and recessions of the neighboring teeth [6,7] and incomplete adaptation of the membrane around irregularly shaped roots, has limited their application in regenerative periodontal surgical procedures.

Enamel matrix protein derivatives (EMD) are the most largely evaluated in both pre-clinical [8] and clinical models, mainly composed of amelogenins, with smaller amounts of other non-amelogenin components, such as tuftelin, ameloblastin and enamel proteases [9]. EMD is a biologically active compound that, once applied on a denuded root surface, starts a cascade of biological events, such as enhanced attraction and migration of mesenchymal cells, their attachment to the root surface [10] and, differentiation into cementoblasts, PDL fibroblasts and osteoblasts. Enamel proteins enhance gene expression responsible for protein and mineralized tissue syntheses in PDL cells [11]. This process may finally lead to reconstitution of the periodontal apparatus.

Application of EMD during reconstructive periodontal surgical therapy enhanced the outcome in respect to clinical attachment level (CAL) gain, probing pocket depth reduction, and new bone formation, compared with open-flap debridement and/or modified Widman flap [6,7,12,13,14,15].

Due to a lesser gingival recession, EMD treatment seems indicated for aesthetic regions. EMD treatment presents less patient morbidity than GTR as membrane exposure occurs in the vast majority of cases treated with GTR, while only few complications occur in EMD treated sites. EMD is a valuable treatment alternative for treating multiple proximal defects without reducing the blood nourishment of the flap leading to extensive membrane exposure. Periodontal regenerative surgery with GTR seems questionable in suprabony defects with horizontal bone loss [16,17], however, EMD application may enhance treatment outcome in these defects [18,19,20,21]. Enhanced clinical wound healing rates following EMD treatment may be appreciated. EMD improved oral mucosa incisional wound healing by promoting the formation of blood vessels and collagen fibres in the connective tissue [22]. The increase in soft-tissue density was faster following EMD application compared to the access flap [23].

EMD enhances gingival fibroblasts proliferation [24,25,26,27,28,29] and positively affects the inflammatory and healing responses by different cellular mechanisms [30,31,32].

## 2. Soft Tissue Considerations

Soft tissue management is one of the most important factors for successful outcomes of periodontal reconstructive surgical treatments. Initially, flap designs were based on conventional periodontal procedures. Later, techniques evolved towards soft tissue preservation to achieve and maintain passive primary closure together with optimal wound stability over the regenerative materials, which is critical especially during the initial healing stages [33,34].

The single flap approach provides access to the surgical site by elevation of a single, either buccal or lingual/palatal full-thickness flap [35,36,37,38,39,40]. The interproximal supracrestal gingival tissues are left intact, allowing for easy flap repositioning with stable primary wound closure. Increased post-surgery gingival recession usually occurs where deep intraosseous are associated with buccal dehiscence defects. The combination of a bioactive agent and a graft material together with a single flap approach may limit postoperative gingival recession [35,36,37,38,39,40].

## 3. Combination Therapies

Combination therapy refers to the simultaneous application of various periodontal reconstructive treatment alternatives to obtain an additive effect. This approach may lead to the assemblage of different regenerative principles, such as conductivity and inductivity, space provision and wound stability, matrix development and cell differentiation.

EMD alone, as a single therapy, may be applied mainly in narrow defects with a prevalent three-wall morphology or in well-supported two-wall defects, biomaterials provide soft tissue support, especially in non-self-contained defects. A large access flap may not provide proper wound stability, which may be achieved with barriers or fillers, combinations of barriers and fillers, or combinations of amelogenins and fillers. The combination of a graft biomaterial with biological agents, including EMD, may reduce the post-surgery recession following surgical treatment accessed with the single flap approach [41].

In most types of defects, application of bone grafting material together with EMD led to additional clinical improvements in CAL gain and PD reduction compared with those obtained with EMD alone [42,43,44].

Periodontal regeneration is the full reconstitution of the lost periodontal support; therefore, application of non-resorbable biomaterials (such as most xenografts) will not lead to true periodontal regeneration.

## 4. Free Connective Tissue Grafts in Periodontal Regenerative Procedures

Another type of combination therapy is the application of autogenous connective tissue grafts during periodontal reconstructive treatment with EMD [18,19,45]. Histological evaluation of combining a connective tissue graft with EMD in humans has shown varying results, including formation of new cementum, new attachment, and new bone formation after treatment [46,47]. EMD has an enhancing effect on gingival fibroblasts, by increasing up to two-fold, both their proliferation and amount of matrix produced by these cells [24,25,26,27,28,29] and positively affects the inflammatory and healing responses by different cellular mechanisms [30,31,32]. Thus, besides the possible periodontal regeneration induction on the denuded root surface, EMD will also enhance the vitality of the free connective tissue graft. During periodontal reconstructive surgery, in cases with minimal amounts of keratinized tissue and in thin periodontal biotypes, a connective tissue can be applied after EMD application onto the denuded root surfaces [20]. This procedure is intended to reduce post-operative gingival recession and increase the gingival dimensions in the area. The beneficial effect of CTG may partly reside in the increase in gingival thickness providing support for the buccal flap. Thick gingival tissues show greater resistance to recession due to surgical trauma and tissue remodelling following different surgical procedures, including regenerative surgery. It may also be speculated that the conversion from a thin to a thick phenotype may have a beneficial effect on the long-term stability of the gingival profile, since thick biotypes were shown to be less prone to developing gingival recessions [48,49].

## 5. Materials and Methods

**Figure 1:**
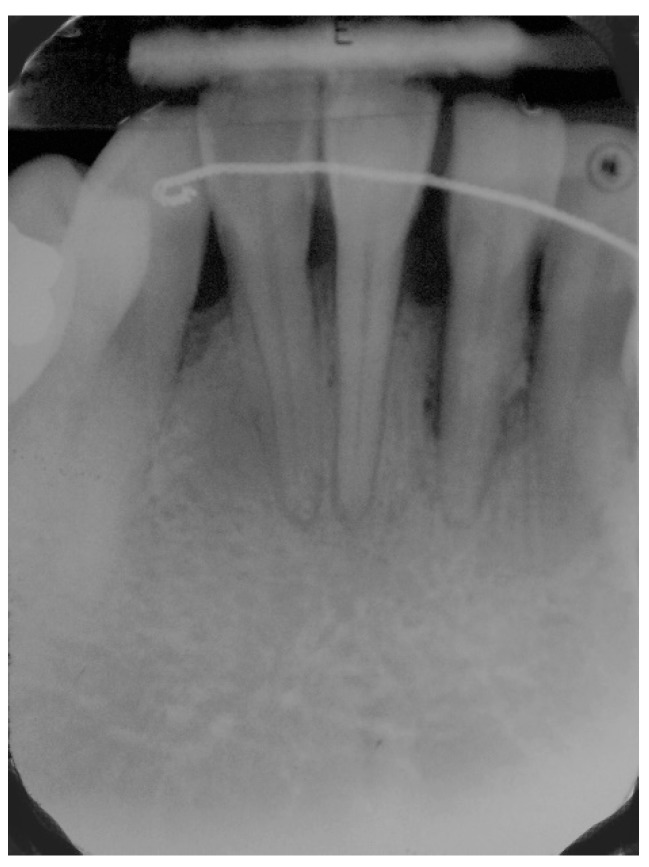
Pre-operative radiograph of lower anterior area showing reduced bone support, with mainly horizontal bone loss.

**Figure 2:**
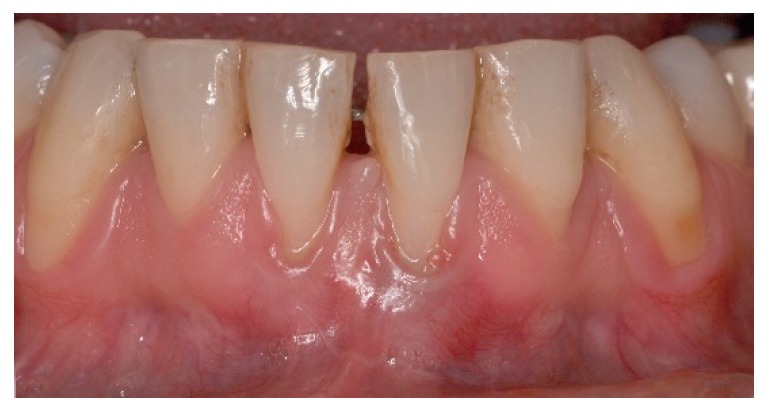
Pre-operative aspect of lower incisors presenting thin gingival biotype, gingival recession, minimal attached and keratinized gingiva width and muscle and frenum pull.

**Figure 3:**
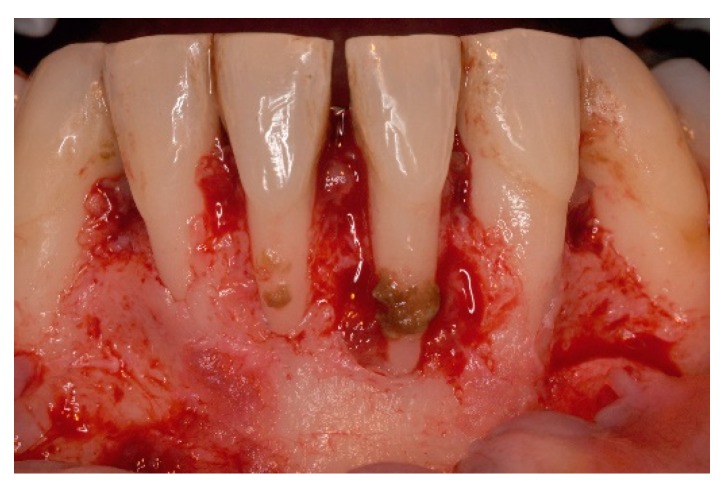
Approach was achieved by raising a single buccal flap, calculus on the denuded root surfaces and large loss of periodontal support are evident.

**Figure 4:**
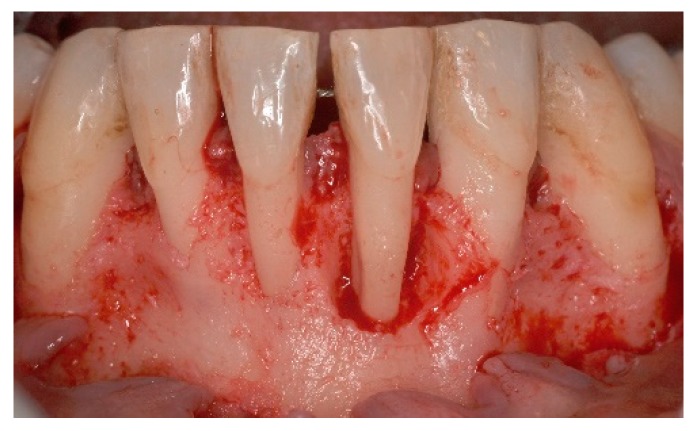
Intra operative aspect, all four lower incisors present extensive root exposure with advanced bone loss, mainly horizontal.

**Figure 5:**
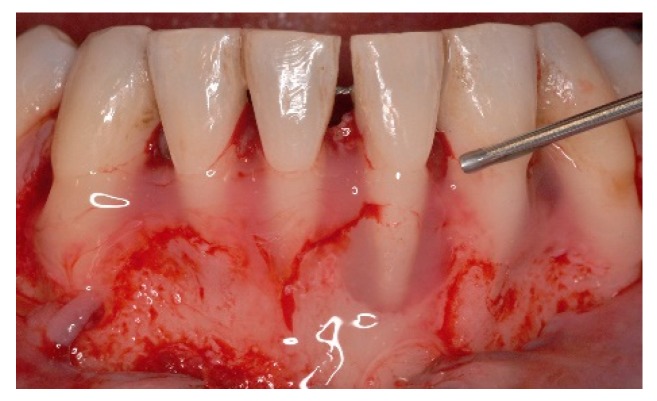
Following root surface conditioning with EDTA gel in a neutral pH for 2 min, enamel maytix proteins derivative gel (EMD) was applied onto denuded root surfaces.

**Figure 6:**
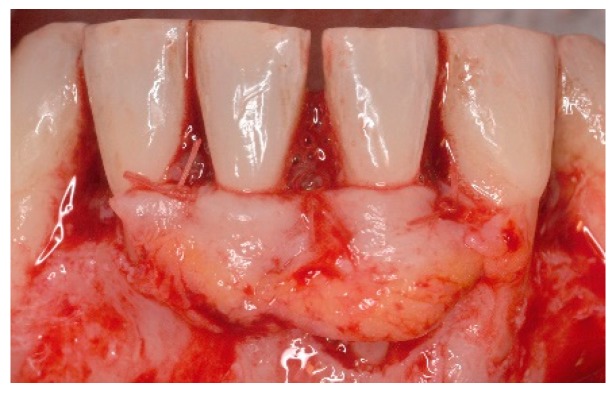
Following EMD application a connective tissue graft was retrieved from the patient’s palate and secured covering the denuded root surfaces.

**Figure 7:**
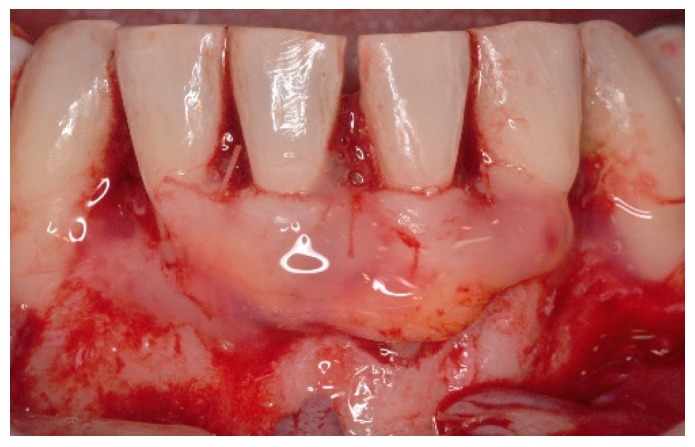
EMD was applied also on top of the soft tissue graft.

**Figure 8:**
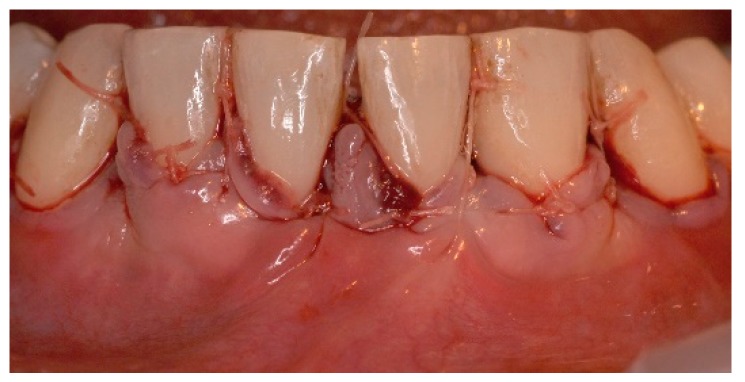
The single buccal flap was coronally displaced and sutured. Tenting sutures were also placed coronally to the contact area to further stabilize the buccal tissues.

The present report is a retrospective evaluation of a surgical procedure performed according to indications. This report includes a series of cases where a modified approach for treatment of severe periodontally involved lower incisors, presenting with thin gingival biotype, gingival recession, minimal attached and keratinized gingiva width and muscle and/or frenum pull, was performed. All cases were treated with a combination therapy consisting of a single buccal access flap, root conditioning, EMD application on the denuded root surfaces and an autogenous free connective tissue graft (Figure 1, Figure 2, Figure 3, Figure 4, Figure 5, Figure 6, Figure 7 and Figure 8). All patients gave proper informed consent agreeing that the data and clinical evidence be made public through publishing provided their identity was not revealed.

## 6. Results

**Figure 9:**
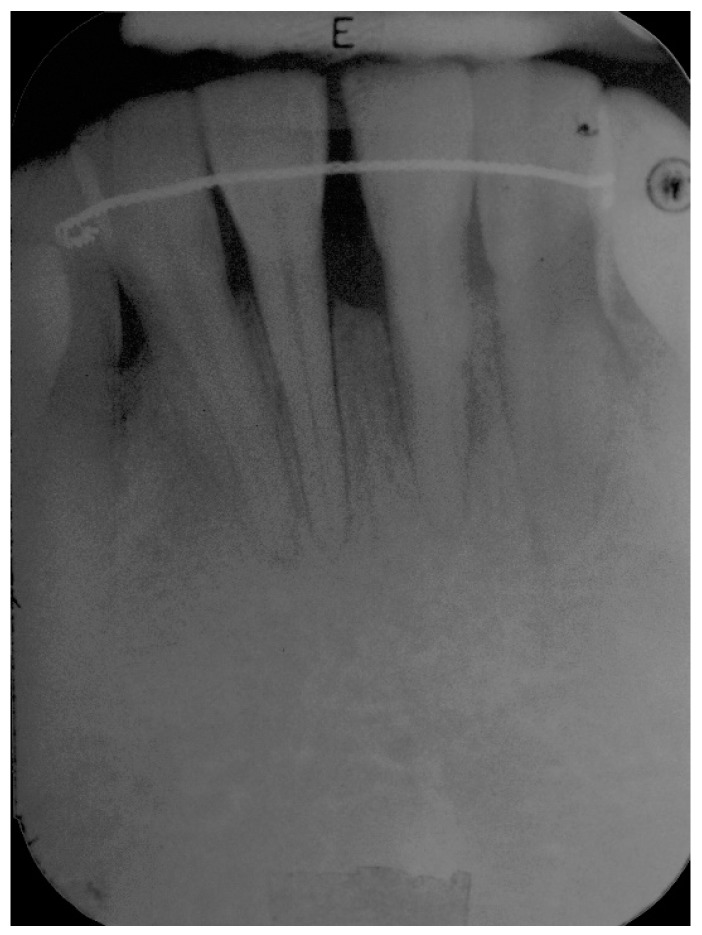
Six-months post-operative radiograph, improved bone support, especially around the central left incisor can already be appreciated.

**Figure 10:**
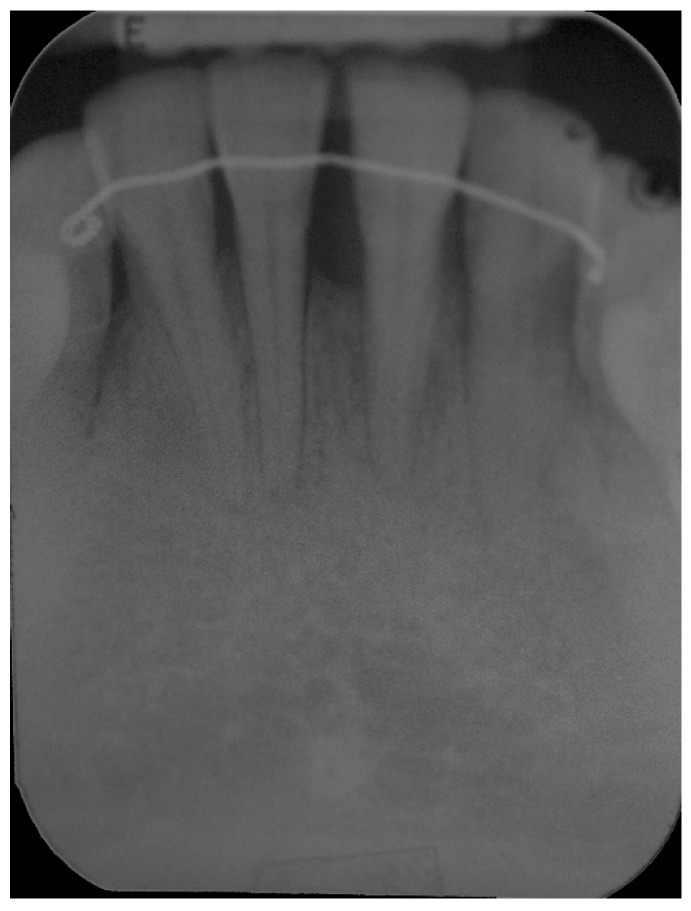
One-year post-operative radiograph, improvement in bone support, especially around the central left incisor is evident, compared to the pre-operative x-ray, the bone defect around the left central incisor has largely been reduced.

**Figure 11:**
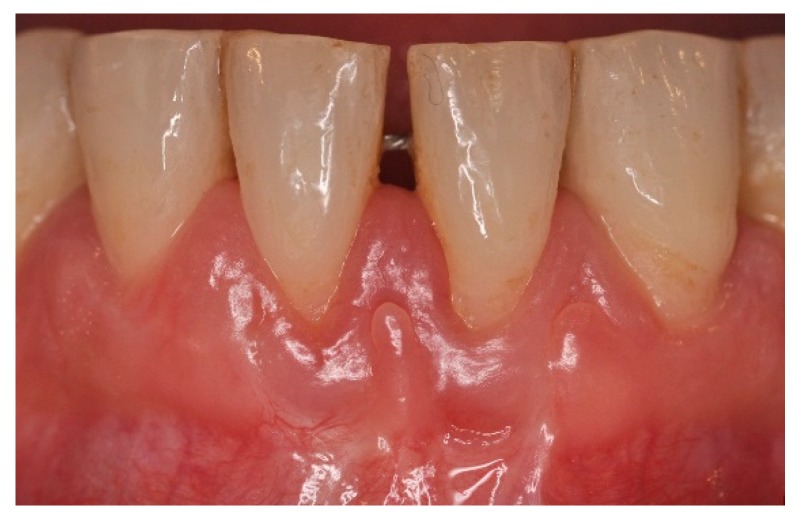
One-year post-operative aspect of lower anterior area. Note gingival aspect, with minimal recession compared to pre-operative aspect, increased attached and keratinized gingival width, with no frenum and muscle pull.

**Figure 12:**
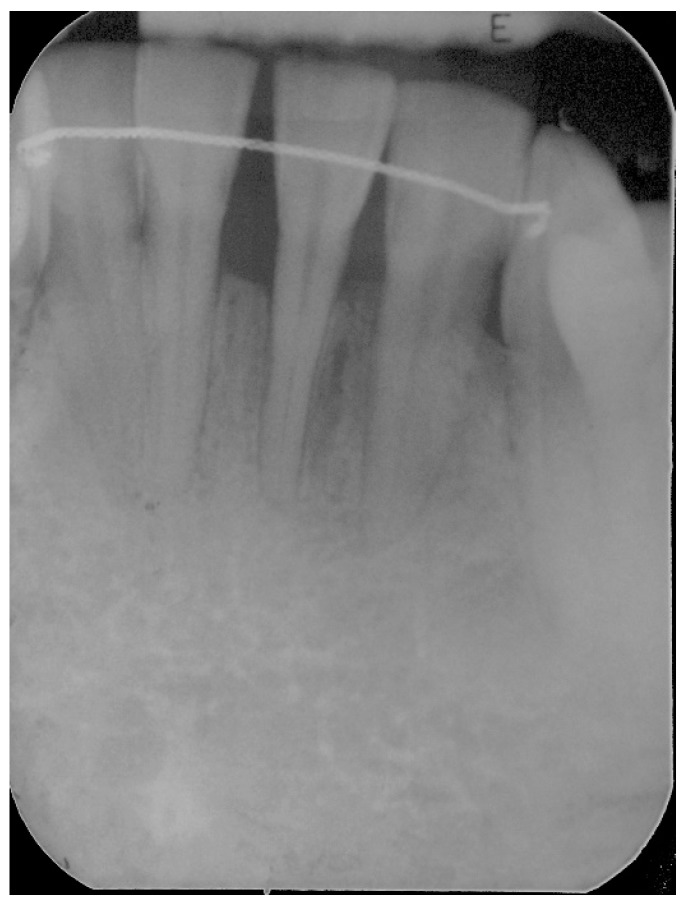
Two-year post-operative radiograph, stable results compared to the one-year situation are evident.

**Figure 13:**
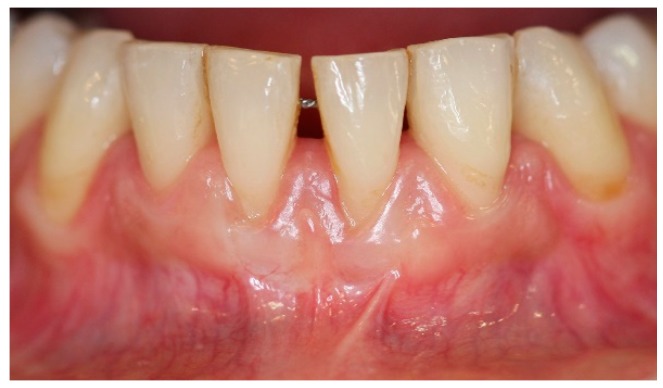
Two-year post-operative aspect of lower anterior area. Note improved gingival aspect, a certain degree of root coverage together with increased attached and keratinized gingival width.

**Figure 14:**
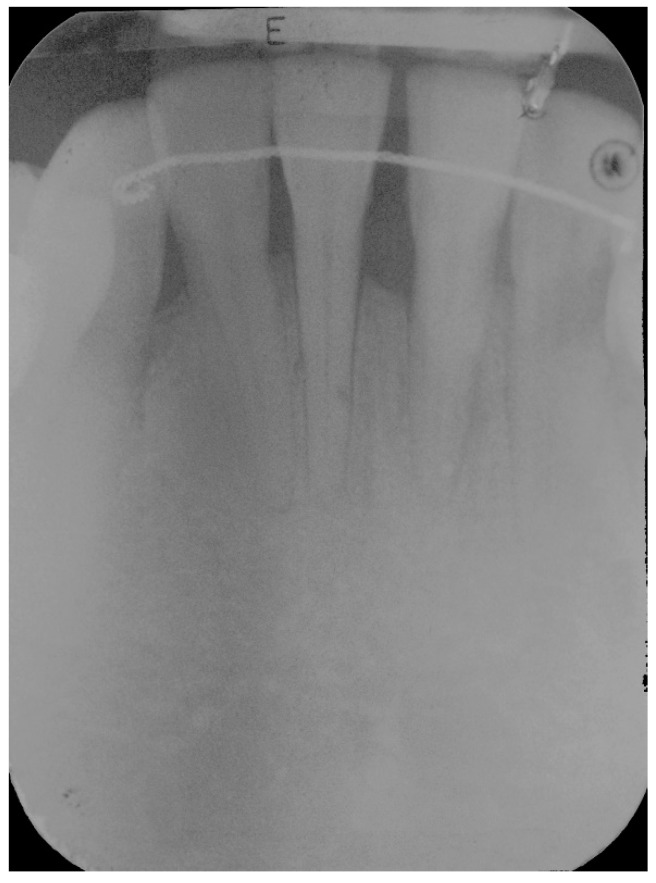
Three years postoperative radiograph, stable results over time can be appreciated.

**Figure 15:**
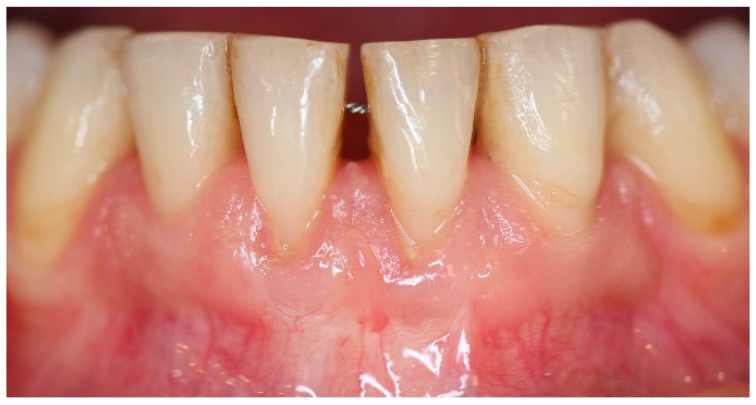
Three-year postoperative aspect of lower anterior area shows stable results over time.

Clinical and radiographic outcomes were consistently satisfactory leading to probing depth reduction, clinical attachment gain minimal gingival recession, increased attached and keratinizing gingival width, with no frenum and muscle pull together with radiographic bone fill of the defects. Provided there was good supportive periodontal therapy, results were stable for long periods (Figure 9, Figure 10, Figure 11, Figure 12, Figure 13, Figure 14 and Figure 15).

## 7. Discussion

In the present report, the single flap approach was combined with an autologous soft tissue graft [48,49] in the lower anterior area. Improved clinical outcomes in terms of both defect resolution, reduction of postoperative gingival recession (or even root coverage) and increase in gingival dimensions in addition to a substantial CAL gain especially for deep intraosseous lesions associated with buccal bone dehiscences, as well as challenging intraosseous defects associated with Miller’s class IV gingival recession, have been reported [20,48,49,50]. Varying degrees of gingival recession are usually appreciated following periodontal surgical treatment; the present approach lead to limited or no post-operative recession. Although the present procedure could also be applied in aesthetic areas to reduce gingival recession following treatment, in the present study it was only applied in the lower anterior area with minimal aesthetic relevance.

The adjunctive use of a CTG unavoidably results in a more technically demanding procedure, and increases the intra- and post-operative morbidity due to the need for an additional surgical site for graft harvesting. The addition of connective tissue grafts to periodontal regenerative surgical procedures seems to be particularly beneficial at defects with thin gingival tissues and severe buccal bone dehiscence, usually in the lower anterior area, however, it is of limited relevance in thick biotypes and shallow buccal dehiscences.

## 8. Conclusions

The combination therapy for reconstructive periodontal treatment in the lower anterior area was able to successfully treat severe periodontally involved lower incisors presenting with thin gingival biotype, gingival recession, minimal attached and keratinized gingiva width and muscle and/or frenum pull. The outcomes showed probing depth reduction, clinical attachment gain minimal gingival recession, increased attached and keratinizing gingival width, with no frenum and muscle pull together with radiographic bone fill of the defects.
